# Hyperpolarized MRI theranostics in cancer

**DOI:** 10.3389/fonc.2025.1693853

**Published:** 2025-11-11

**Authors:** Koya Yamashita, Zhilei Zhao, Keita Saito, Yoichi Takakusagi

**Affiliations:** 1 Institute for Quantum Life Science, National Institutes for Quantum Science and Technology (QST), Chiba, Japan; 2 Center of Quantum Life Science for Structural Therapeutics (cQUEST), Chiba University, Chiba, Japan

**Keywords:** hyperpolarized MRI, theranostics, cancer, metabolism, radiotherapy

## Abstract

Hyperpolarized magnetic resonance imaging (MRI) has emerged as a transformative tool in cancer diagnostics, enabling real-time, non-invasive assessment of tumor metabolism. By employing hyperpolarized molecular probes, such as [1-^13^C]pyruvate, energy metabolism and metabolic changes associated with malignancy in tumors can be visualized, providing key insights into tumor aggressiveness, heterogeneity, and treatment response. In addition to their preclinical and clinical applications in cancer diagnostic imaging, some molecular probes can be used as potentiators of cancer therapy. This perspective article explores the potential use of hyperpolarized magnetic resonance spectroscopic imaging (MRSI) in conjunction with cancer treatment. Notably, the direct application of hyperpolarized molecular probes immediately after imaging to enhance DNA-targeted cancer therapies, including chemotherapeutic drugs and radiotherapy, is termed “hyperpolarized MRI theranostics in cancer.” In this novel approach, metabolic and physiological intratumoral changes induced by biomolecular probes are used to enhance the efficacy of subsequent therapeutic interventions. Additionally, future prospects for advancements in oncology enabled by hyperpolarized MRI are discussed.

## 1 Introduction

Cancer remains a leading cause of mortality worldwide, necessitating the development of advanced therapeutic strategies (1). Clinically, four major cancer treatment strategies are employed: surgical resection, chemotherapy, immunotherapy, and radiotherapy. Chemotherapeutic agents developed to target cancer cells include DNA cross-linking compounds, topoisomerase inhibitors, mitotic inhibitors, and metabolic inhibitors (2, 3). Molecular targeted agents such as imatinib markedly improve chronic leukemia treatment outcomes (4). Similarly, multi-kinase inhibitors such as sunitinib extend progression-free survival in metastatic renal cell carcinoma (5). Advancements in antibody engineering have significantly enhanced the effectiveness of therapeutic antibodies, molecular targeted therapy, and immunotherapy to significantly improve cancer treatment (6).

Radiotherapy is a non-invasive strategy for eradicating cancer cells using high-energy beams with a localized dose distribution. Ionizing radiation induces DNA damage by interacting with DNA and producing free radicals, resulting in cell death. Its efficacy is limited by the hypoxic tumor microenvironment (TME) (i.e., the oxygen effect) (7); however, the use of high-linear energy transfer particles (e.g., α-particles and heavy ion particles) can overcome this radioresistance (8). Some forms of radiation-induced cell death, such as immunogenic cell death, stimulate anticancer immunity, leading to radiotherapeutic effects even in distant, non-irradiated tumors (e.g., metastatic lesions), a phenomenon termed the abscopal effect (9–12). Furthermore, immune checkpoint inhibitors can enhance radiation-induced anti-tumor immunity (13, 14), supporting the use of strategies combining radiotherapy with immunotherapy (15, 16).

Precise radiation dose distribution requires the use of imaging techniques to visualize the tumor location, including computed tomography (CT), positron emission tomography (PET), and magnetic resonance imaging (MRI). Combining diagnostic imaging with radiation exposure, such as in image-guided radiotherapy (IGRT), tomotherapy, and MRI-guided linear accelerators (MR-LINACs), markedly enhances tumor targeting accuracy (17–19). Additionally, pharmaceutical imaging agents are also employed therapeutically, an approach termed theranostics (theragnostics), which combines diagnosis and therapy. In nuclear medicine, companion diagnostics, particularly in PET imaging, have been adopted as a theranostic approach. The susceptibility of patients to radioligand therapy can be evaluated using radiodiagnostics through the accumulation of tumor-avid molecules or antibodies labeled with radioisotopes, such as ^18^F and ^68^Ga, followed by treatment with similar drugs labeled with other radioisotopes, such as ^177^Lu and ^225^Ac (20, 21).

Recent multiomics analyses have revealed metabolic hallmarks associated with malignancy (22–24). Traditional imaging modalities often fail to provide comprehensive metabolic information crucial for effective cancer management, underscoring the importance of functional imaging. Hyperpolarized MRI enables real-time, non-invasive assessment of tumor metabolism by employing hyperpolarized molecular probes such as [1-^13^C]pyruvate (25). Unlike PET, a widespread imaging technique that visualizes metabolic function based on probe uptake and accumulation (e.g., ^18^F-FDG), hyperpolarized MRI enables assessment of the enzymatic reaction of the probe (25). Thus, PET and hyperpolarized MRI provide distinct yet complementary metabolic information, and both modalities can be used for elucidating metabolic functions in cancer.

Beyond the diagnostic applications of ^13^C-probes in hyperpolarized MRI, several studies have indicated that hyperpolarized MRI probes show promise for enhancing therapeutic efficacy. In this perspective article, we introduce the novel concept of “hyperpolarized MRI theranostics in cancer,” which is the direct application of hyperpolarized molecular probes for potentiating cancer treatment immediately following imaging. Compared to the above-mentioned PET-based theranostics, this approach utilizes stable isotope-labeled biomolecular probes that induce metabolic and physiological intratumoral changes, generating therapeutic or sensitizing targets. First, cancer metabolism and the basics of hyperpolarized MRI are outlined; then the potential of hyperpolarized MRI probes for theranostics and their future clinical prospects in oncology are explored.

## 2 Cancer metabolism

### 2.1 Metabolism as a hallmark of cancer

The “Hallmarks of Cancer” conceptual framework, originally introduced in 2000, emphasizes the genetic and signaling alterations underlying malignant traits (26). A 2011 update incorporated dysregulated cellular energetics as a core hallmark, recognizing metabolic reprogramming as a fundamental driver of tumorigenesis (22, 23). Recent conceptual advancements have reframed cancer metabolism as a system-level adaptation that extends beyond cell-autonomous processes to encompass dynamic interactions with both the TME and host (27, 28). Metabolic plasticity enables cancer cells to transition between epithelial and mesenchymal states, acquire stem-like properties, and adapt to fluctuating nutrient and oxygen availability through the reprogramming of glucose, glutamine, and lipid metabolism (29, 30).

The tumor-associated microbiome also influences cancer metabolism, with microbial metabolites (e.g., short-chain fatty acids and bile acids) modulating cancer cell signaling, immunity, and therapeutic responses (31, 32). Additionally, metabolic alterations are increasingly linked to the senescence-associated secretory phenotype (SASP), where senescent stromal and immune cells release bioactive factors that reshape TME metabolism, promote immunosuppression, and drive tumor progression (33, 34).

Competition for metabolic resources within the TME further exacerbates immune evasion, as cancer cells consume key nutrients (e.g., glucose and tryptophan) and release immunosuppressive oncometabolites, such as lactate and kynurenine, thereby impairing anti-tumor immunity (35, 36).

### 2.2 Metabolic reprogramming

**Figure 1:**
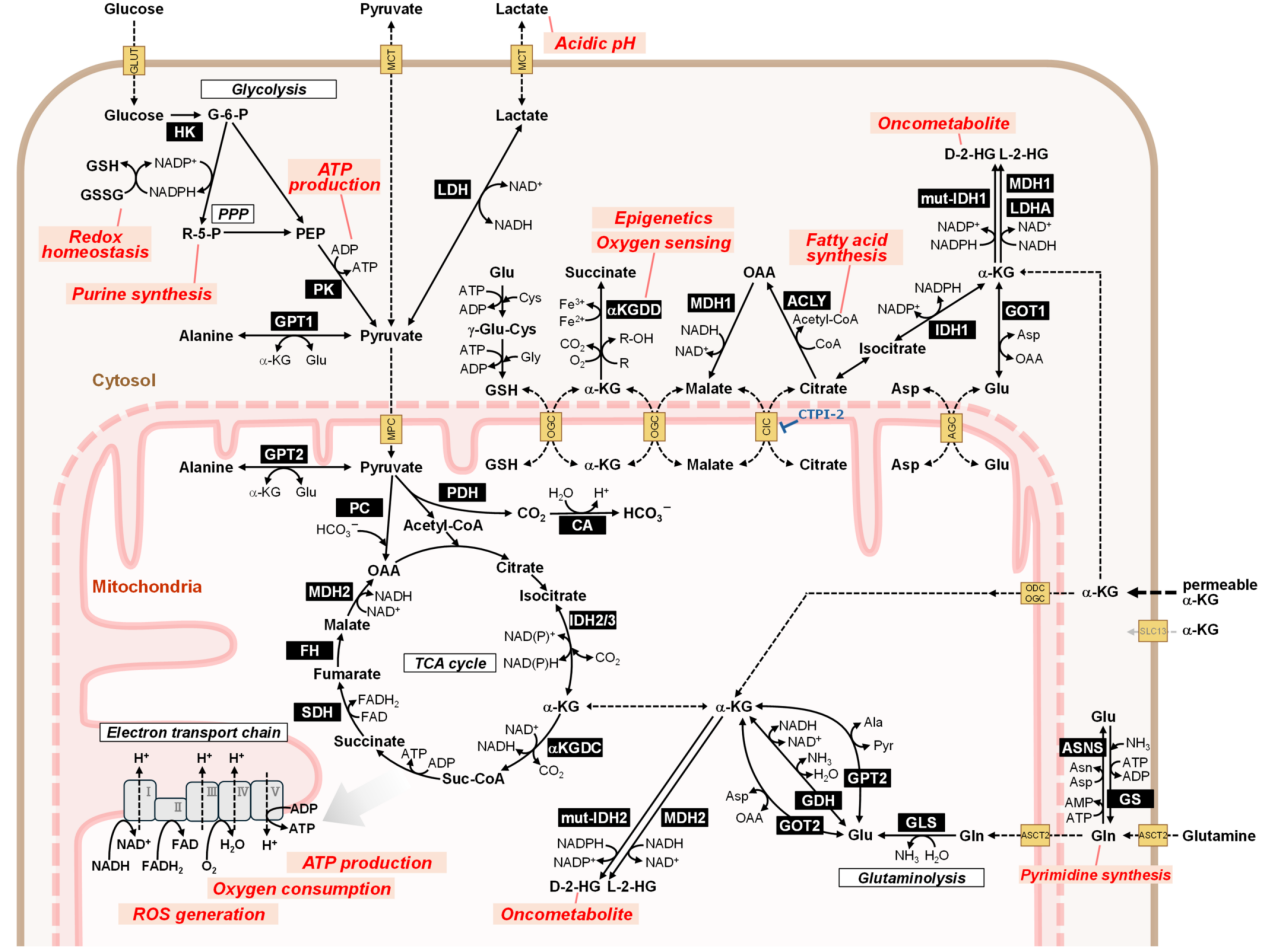
Overview of metabolic pathways relevant to hyperpolarized MRI theranostics in cancer. Metabolic reprogramming is a key hallmark of cancer. Cancer cells exhibit enhanced glucose consumption and rely on glycolysis regardless of oxygen concentration (Warburg effect). The most promising hyperpolarized MRI probe, [1-^13^C]pyruvate, can be used to evaluate metabolic activities in glycolysis (lactate production), the TCA cycle (bicarbonate production), and amino acid synthesis (alanine production). To compensate for the limited pyruvate entry into the TCA cycle owing to enhanced lactate production, cancer cells utilize glutamine via the glutaminolysis pathway. The TCA cycle generates intermediate metabolites for biosynthesis and reduced cofactors, such as NADH and FADH_2_, for ATP production via oxidative phosphorylation in the mitochondrial electron transport chain. *IDH* gene mutations result in the abnormal production of the oncometabolite 2-HG from α-ketoglutarate. Hyperpolarized MRI using α-ketoglutarate can monitor this reaction and thus serve as a probe for *IDH* mutation. Cellular metabolic pathways are coordinated beyond the plasma membrane through transporters. Disrupting this orchestration represents a promising cancer treatment strategy. Metabolic changes triggered by hyperpolarized MRI probes may potentially enhance therapeutic effectiveness. Notably, the metabolites, proteins, and pathways shown are excerpted representatives, and not all participants are illustrated. The names of the enzymes are highlighted with a black background. Biologically important processes are indicated by red characters. 2-HG, 2-hydroxyglutarate; ACLY, ATP citrate lyase; AGC, aspartate-glutamate carrier (SLC25A12/13); α-KG, alpha-ketoglutarate; αKGDC, α-ketoglutarate dehydrogenase complex (2-oxoglutarate dehydrogenase complex, OGDHc, OGDC); αKGDD: α-ketoglutarate dependent dioxygenase (2-oxoglutarate dependent dioxygenase, 2OGDD); Ala, alanine; ASCT2, alanine serine cysteine transporter 2 (ASC transporter 2, SLC1A5); Asn, asparagine; ASNS, asparagine synthetase; Asp, aspartate; CA, carbonic anhydrase; CIC, mitochondrial citrate carrier (SLC25A1); Cys: cysteine; FH, fumarate hydratase (fumarase); γ-Glu-Cys, gamma glutamyl cysteine; G-6-P, glucose-6-phosphate; GDH, glutamate dehydrogenase (GLDH); Gln, glutamine; GLS, l-glutamine amidohydrolase (glutaminase); Glu, glutamate; GLUT, glucose transporter (SLC2A); Gly, glycine; GOT, glutamic-oxaloacetic transaminase (aspartate transaminase, AST); GPT, glutamic-pyruvic transaminase (alanine transaminase, ALT); GS, glutamine synthetase; GSH, reduced form of glutathione; GSSG, glutathione disulfide (oxidized form of glutathione); HK, hexokinase; IDH, isocitrate dehydrogenase; LDH, lactate dehydrogenase; MCT, monocarboxylate transporter (SLC16A); MDH, malate dehydrogenase; MPC, mitochondrial pyruvate carrier (SLC54); mut-IDH, mutated isocitrate dehydrogenase; OAA, oxaloacetic acid; ODC, mitochondrial oxodicarboxylate carrier (SLC25A21); OGC, mitochondrial oxoglutarate malate carrier (SLC25A11); PC, pyruvate carboxylase; PDH, pyruvate dehydrogenase; PEP, phosphoenolpyruvic acid; PK, pyruvate kinase; PPP, pentose-phosphate pathway; R-5-P, ribose-5-phosphate; ROS, reactive oxygen species; SDH, succinate dehydrogenase (respiratory complex II); Suc-CoA, succinyl-CoA; TCA cycle, tricarboxylic acid cycle (citric acid cycle, Krebs cycle).

Metabolic reprogramming is a hallmark of cancer that involves changes in central carbon metabolism, lipid synthesis, and amino acid use to provide the energy, building blocks, and redox balance required for uncontrolled growth (**Figure 1**, 37). Beyond changes in glycolysis, cancer cells rewire the tricarboxylic acid (TCA) cycle and exhibit adaptive amino acid metabolism (38–40). This metabolic reprogramming involves a shift in the glutamine-derived nitrogen flux from anaplerotic pathways to nucleotide biosynthesis (41, 42). This shift is facilitated by increased phosphoribosyl pyrophosphate amidotransferase (PPAT) activity relative to that of glutaminase (GLS1). Higher PPAT/GLS1 ratios are linked to poor prognosis in aggressive cancers such as small-cell lung cancer. Suppressing PPAT significantly reduces tumor growth, highlighting glutamine nitrogen metabolism as a potential treatment target (41). Mutations in driver genes, such as *KRAS* and *TP53*, play a significant role in orchestrating these metabolic changes (43). Environmental factors such as hypoxia and nutrient scarcity further promote metabolic flexibility (44, 45). Moreover, specific oncometabolites, such as 2-hydroxyglutarate (2-HG), in isocitrate dehydrogenase (IDH)-mutant cancers act as epigenetic modifiers and reinforce cancer-promoting programs (43, 46).

### 2.3 Relationship between cancer metabolism and DNA damage/repair in radiotherapy

Ionizing radiation induces radiation responses at the intracellular, intratumoral, and systemic levels. DNA damage activates signaling pathways that regulate DNA repair and cell cycle arrest, ultimately inducing cell death (47). Dynamic metabolic rewiring, which provides cells with metabolites involved in antioxidant defense and DNA repair, occurs in irradiated cells to support the DNA damage response (48, 49). Enhanced glycolysis supplies ribonucleotides for nucleotide synthesis (DNA repair) and NADPH for the reduction of oxidized glutathione via the pentose phosphate pathway (50, 51). Additionally, increased glycolytic capacity results in the acidification of the intratumoral pH through lactate efflux (52, 53), an end product of glycolysis whose production is essential for replenishing NAD^+^ in cancer (54). Activation of the mitochondrial electron transport chain increases ATP production and oxygen consumption following irradiation, and mitochondrial activation and/or dysfunction causes abnormal reactive oxygen species (ROS) production, which disrupts redox homeostasis (55–58). Given that DNA repair is highly energy-intensive, insufficient ATP production is critical for irradiated cells (59–61). Accordingly, considering the hallmarks of cancer and therapy-induced changes, metabolism represents a potential target for sensitizing treatments, including radiotherapy (3).

## 3 Hyperpolarized ^13^C MRI

Hyperpolarized MRI has gained increased attention owing to the need for direct monitoring of cancer metabolism in radiotherapy. Hyperpolarization by dynamic nuclear polarization (DNP) is a promising technique that enhances the magnetic resonance signal by over 10,000-fold (62). This enables hypersensitive NMR spectroscopy and magnetic resonance spectroscopic imaging (MRSI) to directly probe enzymatic activity and metabolic reprogramming, including the Warburg effect. This technique can also assess therapeutic responses to chemotherapy or radiotherapy during the early phase (63–65).

**Figure 2:**
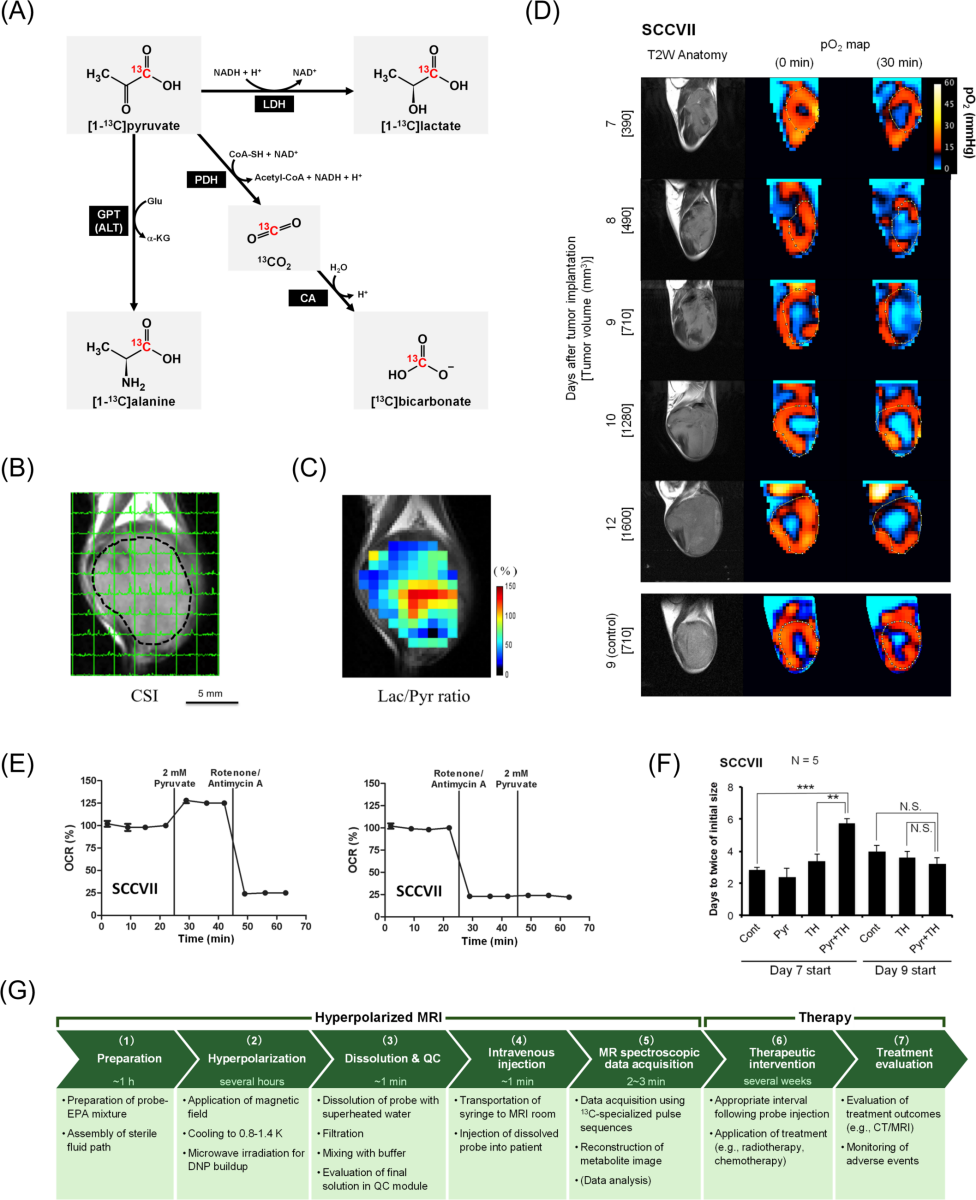
Hyperpolarized MRI of pyruvate metabolism followed by hypoxia induction and its potential for augmenting cancer treatment. Panels **(B–F)** are cited from the indicated references. **(A)** Diagram of [1-^13^C]pyruvate metabolism. [1-^13^C]pyruvate is metabolized to [1-^13^C]lactate, [1-^13^C]alanine, and [^13^C]bicarbonate via ^13^CO_2_. **(B)** Non-invasive evaluation of pyruvate metabolism using hyperpolarized MRI in SCC VII tumor-bearing mice. Each signal peak corresponds to [1-^13^C]pyruvate (right) and [1-^13^C]lactate (left). **(C)** Heatmap of the lactate-to-pyruvate ratio (Lac/Pyr) calculated based on **(B)** overlaid on the anatomical image. Panels **(B, C)** were reproduced from (70) the publication Matsuo M, Kawai T, Kishimoto S, Saito K, Munasinghe J, Devasahayam N, et al. Co-imaging of the tumor oxygenation and metabolism using electron paramagnetic resonance imaging and 13-C hyperpolarized magnetic resonance imaging before and after irradiation. Oncotarget (2018). 9:25089–25100. (doi: 10.18632/oncotarget.25317). Creative Commons CC BY 3.0. **(D)** Non-invasive monitoring of tumor pO_2_ distribution by electron paramagnetic resonance imaging before and 30 min after pyruvate injection in SCC VII tumor-bearing mice. **(E)***In vitro* monitoring of oxygen consumption rates (OCR) under treatment with pyruvate and mitochondrial electron transport chain inhibitors (rotenone/antimycin A). **(F)** Tumor doubling time following treatment with pyruvate and/or the hypoxia-activated prodrug TH-302. TH-302 (100 mg/kg) was intraperitoneally injected 30 min after pyruvate injection. **, *P*<0.01, ***, *P*<0.001. Panels **(D–F)** were reproduced from (76) the publication Takakusagi Y, Matsumoto S, Saito K, Matsuo M, Kishimoto S, Wojtkowiak JW, et al. Pyruvate Induces Transient Tumor Hypoxia by Enhancing Mitochondrial Oxygen Consumption and Potentiates the Anti-Tumor Effect of a Hypoxia-Activated Prodrug TH-302. PLoS One. (2014) 9:e107995. (doi: 10.1371/journal.pone.0107995). Creative Commons CC0. Pyruvate was administered intravenously at a consistent dose (1.15 mmol/kg) across Panels **(B-D)** and **(F)**. **(G)** Workflow of hyperpolarized MRI theranostics for cancer treatment. An example workflow is presented with the approximate time requirements for each step. (1) Preparation for hyperpolarization: A ^13^C-labeled probe and electron paramagnetic agent (EPA) are mixed with a glass-forming agent. The sterile fluid path is assembled. (2) Hyperpolarization in a DNP hyperpolarizer: The probe mixture is inserted into a polarizer, where it is placed under a magnetic field at 0.8–1.4 K. Microwave irradiation is applied to transfer the spin polarization from electrons to ^13^C-nuclei. The time required to achieve sufficient polarization depends on the probe, protocol, and polarizer (e.g., over 2 h for [1-^13^C]pyruvate). During this time, the patient is positioned in the MRI machine, and preliminary scans (e.g., ^1^H anatomy imaging) for localization and calibration are performed. (3) Dissolution and quality control (QC): The hyperpolarized probe is rapidly dissolved in superheated water, filtered to remove EPA, mixed with buffer, and sterile filtered into the administration syringe. The final solution is automatically tested using a QC module to ensure it meets the release criteria (e.g., concentration, pH, sterility, polarization level, residual EPAs, and temperature). (4) Intravenous injection: The syringe is transported to the MRI room, and the hyperpolarized probe is intravenously administered as a bolus injection (e.g., 230 mM, 0.43 mL/kg body weight, 5 mL/s for [1-^13^C]pyruvate). The duration from dissolution to injection is approximately 60 s. (5) MR spectroscopic data acquisition: Data are acquired using pulse sequences specialized for ^13^C MRI. Scanning begins within a few minutes after dissolution to minimize signal loss due to relaxation. Metabolite images are reconstructed from the data. Data analysis (quantification and parameter calculation) provides translatable functional information. If necessary, additional MRI sequences can be performed, such as diffusion-weighted imaging and dynamic contrast-enhanced MRI. (6) Therapeutic intervention: To potentiate efficacy, patients should receive therapies (such as radiotherapy or chemotherapy) an appropriate duration after probe injection (e.g., 30 min for pyruvate-induced hypoxia). Treatment may continue for several weeks depending on the procedure. (7) Treatment evaluation: Treatment outcomes are evaluated using imaging (X-rays/CT/MRI) to assess tumor size reduction or elimination. Adverse events must be monitored during and after treatment.

Molecular probes are central to the functionality of hyperpolarized MRI. These probes, typically labeled with isotopes such as ^13^C or ^15^N, are hyperpolarized to enhance their MRI signals (66). Upon administration, they undergo metabolic transformations within the body, and their conversion products are detected using MRI. For instance, [1-^13^C]pyruvate is typically used to assess glycolytic activity, as it is rapidly converted to [1-^13^C]lactate and [^13^C]bicarbonate in tumors (**Figures 2A–C**) (67–70). The relative concentrations of these metabolites provide insights into the metabolic state of the tumor, which can be indicative of malignancy and aggressiveness. Therefore, hyperpolarized MRI can be used as a non-invasive means to monitor the effects of novel therapeutic agents and evaluate their impact on tumor metabolism.

Among ^13^C-labeled probes, clinical trials have predominantly focused on [1-^13^C]pyruvate, which enables real-time quantification of pyruvate-to-lactate flux (k_PL_) in prostate cancer (NCT04286386), ischemic heart disease (NCT06054516), and primary central nervous system lymphoma (NCT04656431). Several other ^13^C-labeled probes are being explored in clinical trials to provide complementary metabolic and physiological information. For instance, [2-^13^C]pyruvate has been used to evaluate TCA cycle activity and mitochondrial function in prostate cancer (NCT04346225). ^13^C-bicarbonate enables noninvasive imaging of extracellular tumor pH (NCT05851365), while α-ketoglutarate (α-KG) can be used for imaging IDH-mutant tumors through its conversion to 2-HG (NCT05851378). ^13^C,^15^N-urea serves as a metabolically inert perfusion marker that can be used to assess renal and tumor vasculature and co-polarized with pyruvate to simultaneously provide perfusion and metabolic information (NCT06391034) (71). Additionally, ^13^C-fumarate detects necrotic tissue via conversion to malate and can monitor therapeutic responses, with translational studies supporting its clinical applicability (ISRCTN49119680).

## 4 Representative hyperpolarized ^13^C molecular probes and theranostic applications

Hyperpolarized ^13^C-labeled molecules are administered as a bolus injection within a narrow time window (5–20 s) to obtain metabolic images to preserve hyperpolarized ^13^C signals before they decay. Using this standard rapid injection protocol, some molecular probes have been found to induce physiological and pharmacological changes within the TME and enhance therapeutic efficacy against cancers.

### 4.1 Pyruvate

Pyruvate is currently the most promising clinical ^13^C molecular probe and is used to evaluate the metabolic capacity of both the glycolysis pathway and TCA cycle and monitor tumor progression and treatment response (72–74). Although hyperpolarized [1-^13^C]pyruvate provides valuable metabolic information, intravenous bolus pyruvate injection induces transient hypoxia that lasts for several hours (75–77). Pyruvate administration results in increased mitochondrial oxygen consumption, which contributes to an acute decrease in tumor tissue oxygen pressure (pO_2_) (**Figures 2D, E**) (76, 77). For instance, a mouse squamous cell carcinoma (SCC) model exhibited resistance to radiotherapy within 5 h of pyruvate injection attributable to this induced hypoxia (75). Notably, these pharmacological effects only occur in living individuals (*in vivo*), as they depend on the interplay between cancer cells and TME, and are unlikely to be fully recapitulated *in vitro* due to the absence of intratumor vasculature. This underscores that the direct analysis of biological processes *in vivo*, including energy metabolism, is crucial for advancing medical technology.

Although the physiological effects of pyruvate on the TME must be considered in radiotherapy, they can also be exploited in hypoxia-targeted treatment strategies such as hypoxia-activated prodrugs (HAPs). Evofosfamide (TH-302) can be activated depending on the oxygen concentration (<76 mmHg), particularly under severe hypoxic conditions (<10 mmHg) such as in the TME, and releases a warhead to crosslink double-stranded DNA, resulting in cell death (78–81). Pyruvate-induced hypoxia enhances the anti-tumor effects of TH-302 in mouse SCC (**Figure 2F**), human colon cancer, and human pancreatic ductal adenocarcinoma models (76, 77). Because TH-302 exhibits minimal toxicity in aerobic tissues (76), a potential theranostic strategy could involve combining transient tumor hypoxia (induced by hyperpolarized [1-^13^C]pyruvate MRI) with subsequent HAP administration.

Moreover, the combination of TH-302 and X-ray irradiation yields superior outcomes compared to either treatment alone (81–83). Fundamentally, radiotherapy targets normoxic lesions but not hypoxic fractions; however, TH-302 functions in the opposite manner. Thus, both therapeutic strategies are complementary and can function synergistically. Additionally, TH-302 reduces intratumor oxygen levels (i.e., reoxygenation) by eliminating hypoxic tumor cells and lowering oxygen demand, thereby enhancing the effectiveness of subsequent ionizing radiation (83). Overall, we anticipate that the theranostic approach can be conducted as follows: First, metabolic diagnosis is performed using hyperpolarized [1-^13^C]pyruvate, which induces transient hypoxia. This can be exploited to potentiate the activity of HAPs administered 30 min after pyruvate injection, which eliminate tumor cells in the hypoxic fraction. Finally, 1–2 days after HAPs administration, radiotherapy can be used to target normoxic tumor cells, thereby complementing HAPs and leveraging their tumor reoxygenation activity. For clinical applications, appropriate combination therapy protocols must be developed. For instance, pyruvate + HAP administration only once on the imaging day may be insufficient for treatment. Therefore, the treatment days required to eradicate hypoxic cells and achieve reoxygenation prior to radiotherapy need to be established.

### 4.2 Alpha-ketoglutaric acid

Alpha-ketoglutarate, known as 2-oxoglutarate, is a TCA cycle intermediate produced from glutamate via glutamate dehydrogenase (GDH), thus linking the glutaminolysis pathway with the TCA cycle following glycolysis (84, 85). In hyperpolarized MRI, α-KG is used for probing *IDH1* mutations (e.g., glioma) by monitoring the conversion of α-KG into the oncometabolite 2-HG (86–88). 2-HG has multiple roles in tumor progression and immune regulation (46, 89). α-KG-dependent dioxygenase (αKGDD), also known as 2-oxoglutarate-dependent dioxygenase (2OGDD), is a key target of 2-HG (90). The αKGDD superfamily comprises enzymes with diverse biological functions, including oxygen sensing, epigenetic regulation, and extracellular matrix formation (91, 92). αKGDD enzymes require α-KG, divalent iron (Fe^2+^), and oxygen as co-substrates and produce hydroxylated substrates, succinate, trivalent iron (Fe^3+^), and CO_2_. Dysregulation of αKGDD has been observed in various cancers (91, 93). Considering the importance of 2-HG, hyperpolarized α-KG MRI provides valuable insights for precision medicine, and derivatives with enhanced membrane permeability (e.g., diethyl-α-KG) enhance its feasibility (94).

More recently, α-KG has been identified as a radiosensitizer for lung cancer treatment in combination with CTPI-2, an inhibitor of mitochondrial citrate carrier (SLC25A1; CIC), whereas α-KG alone had no pharmacological effects (95). Although CTPI-2 alone can increase d-2-HG levels and act as a radiosensitizer by modulating DNA repair (96), the combination of α-KG and CTPI-2 significantly enhanced d-2-HG levels and radiosensitivity. This effect did not solely depend on d-2-HG, suggesting that metabolic alterations such as NAD^+^/NADH imbalance also contribute. Collectively, these findings demonstrate that hyperpolarized α-KG probes can be used as theranostic agents to potentiate CTPI-2 efficacy in specific cancers following metabolic imaging.

αKGDD activity is also inhibited by TCA cycle intermediates, including succinate and fumarate (91). Solute carriers (SLC) on the mitochondrial membrane play key roles in transporting TCA intermediates to the cytosol (97, 98). Therefore, metabolic modulation, which impacts the production, consumption, and transportation of TCA intermediates, can attenuate oncogenic αKGDD activity and modulate biosynthesis and the ATP/ADP and NAD(P)^+^/NAD(P)H balance (97, 99). Notably, the cell-permeable derivative 1-trifluoromethyl benzyl-α-KG can disrupt cellular energy metabolism and induce cell death under hypoxic conditions (100). These observations further support combining mitochondrial SLC inhibitors with cell-permeable α-KG to modulate metabolism and thus enhance therapeutic efficacy.

Overall, the theranostic workflow can be summarized as follows: First, metabolic diagnosis is performed using hyperpolarized α-KG, followed by CTPI-2 administration, and finally, radiotherapy is applied. Preclinical studies have explored administering drugs 2 h before radiotherapy both *in vitro* and in an *in vivo* chick embryo chorioallantoic membrane model (95); however, the feasibility and optimal protocol, including timing after imaging and radiotherapeutic conditions, require further investigation in animal experiments.

A workflow diagram of hyperpolarized MRI theranostics for cancer is illustrated in **Figure 2G**.

## 5 Discussion

### 5.1 Metabolism as a therapeutic target

Insights into the systemic role of metabolism have revealed novel therapeutic opportunities, including targeting metabolic dependencies through enzymes such as lactate dehydrogenase (LDH) and GLS, disrupting metabolic plasticity to inhibit adaptive fuel switching under stress, and combining metabolic inhibitors with immunotherapies such as anti-PD-1 to enhance immune responses. By transcending the boundaries of a single hallmark, metabolic reprogramming integrates genetic, epigenetic, and environmental factors from cancer initiation and progression to therapeutic resistance, holding significant promise for next-generation therapies.

### 5.2 Advantages of radiotherapy in hyperpolarized theranostics

The systemic effects of hyperpolarized MRI probes remain unclear, although adverse events are rare in clinical metabolic imaging (101). Nonetheless, some metabolic modulation may occur even in normal tissues, potentially enhancing or triggering adverse effects when combined with therapeutic interventions. Thus, it is anticipated that tumor-selective treatment strategies, such as HAPs and radiotherapy, are better suited for hyperpolarized MRI theranostics. To minimize radiation exposure to the surrounding healthy organs, radiation beams are generally targeted at the tumor region from multiple angles, with doses adjusted to match tumor shape, in techniques known as intensity-modulated radiation therapy (IMRT) and volumetric modulated arc therapy (VMAT) (17). Delivering tumor-localized radiation can minimize the risk of adverse events in the surrounding organs while leveraging the physiological modulation accompanied by hyperpolarized MRI. To this end, MR-LINAC is a promising radiotherapy platform owing to its high contrast that enables soft tissue discrimination and precise dose delivery through motion management during irradiation (102, 103). Performing, hyperpolarized MRI using MR-LINAC is an attractive strategy; however, it faces challenges regarding limited magnetic field strength (1.5 T in Elekta Unity MR-LINAC vs. 3 T in most human hyperpolarized MRI studies) (101, 103), spatial positioning accuracy, and the need to update MR-LINAC systems to support multinuclear imaging.

### 5.3 Comparison of PET-based and hyperpolarized MRI theranostics

Hyperpolarized MRI theranostics differs substantially from PET-based radiotheranostics: 1) Hyperpolarized MRI employs stable isotope (^13^C)-labeled probes, thus avoiding the administration of radioactive compounds, but requires an external radiation source or DNA-damaging agent for therapy; 2) therapeutically, hyperpolarized molecular probes induce physiological and metabolic changes that potentiate therapeutic efficacy; 3) radioligand imaging predicts the effectiveness of the subsequent radioligand therapy, whereas the MRI results and subsequent treatment outcomes are not necessarily correlated in hyperpolarized MRI theranostics. As better optimized post-imaging interventions are developed, the importance of this method will increase; and 4) since synthetic radioligands accumulate by targeting cell surface proteins or enhanced permeability and retention (EPR) effects, off-target effects may occur depending on the drug-delivery systems. Conversely, ^13^C-labeled biomolecular probe distribution relies on perfusion and cellular uptake, which is less organ selective and may require tumor-targeted therapeutic interventions.

### 5.4 Limitations of current hyperpolarized MRI theranostic approaches

The concept of hyperpolarized MRI theranostics is based on the chick embryo model (α-KG) and animal experiments (pyruvate), whose conditions differ from those of humans. For example, the dose of [1-^13^C]pyruvate used in hyperpolarized MRI experiments in mice (1.15 mmol/kg; 300 μL/mouse; 96 mM) (75) is over 10 times higher than doses required for humans (0.10 mmol/kg; 0.43 mL/kg; 230 mM) (68). As there is no evidence for pyruvate-induced hypoxia or radiosensitization from using the combination of α-KG and CTPI-2 in humans, the clinical feasibility of hyperpolarized MRI theranostics warrants further investigation.

Several clinical trials have investigated the use of TH-302 as a single agent or in combination with chemotherapeutic drugs; however, it has not yet been approved by the Food and Drug Administration (FDA). Other HAPs have similarly encountered challenges in clinical trials (104). Therefore, it is worth investigating whether these drugs exhibit significant efficacy under pyruvate-induced severe hypoxic conditions, as other HAPs beyond TH-302 may benefit from this strategy.

### 5.5 Future prospects for hyperpolarized MRI theranostics

Currently, hyperpolarized MRI faces several barriers to clinical translation, including regulatory hurdles, high costs, and reproducibility challenges (101, 105, 106). The widespread adoption of hyperpolarized MRI is crucial for realizing its theranostic applications. The scarcity of preclinical and clinical facilities equipped with dissolution DNP polarizers represents a major limitation; thus, increasing this infrastructure is crucial. The development of dissolution DNP techniques that enable transportable hyperpolarized probes will substantially increase clinical use and accessibility (107, 108). Furthermore, the development of rapid and cost-effective hyperpolarization techniques such as parahydrogen-induced polarization (PHIP) (109) or signal amplification by reversible exchange (SABRE) (110–112) is crucial for maximizing scalability (113, 114).

For the clinical application of hyperpolarized MRI theranostics, the probe must be approved for both imaging and therapeutic purposes. Research on their use as imaging probes in humans has already been initiated (101, 115). The quality of the probe must be guaranteed, and its preparation procedures resemble those of radiopharmaceuticals, requiring an on-site pharmacy kit (116). For use as a therapeutic sensitizer, additional clinical trials are required. Once the probe has been approved and clinically used for imaging, drug repositioning to expand its indications may streamline and accelerate therapeutic approval (117). For a new probe to obtain approval equivalent to an Investigational New Drug prior to clinical trials, preclinical studies confirming safety and efficacy are mandatory. ^13^C atoms on these probes enable preclinical evaluation of their pharmacokinetics.

Among hyperpolarized MRI probes, pyruvate is anticipated to lead in therapeutic applications. However, other probes evaluated in clinical trials (introduced in Section 3) also demonstrate therapeutic potential. Furthermore, previously developed probes, such as dehydroascorbic acid (redox evaluation) (118, 119), have potential application in this field in combination with radiotherapy, which warrants further exploration. Mechanistically, since hyperpolarized MRI targets the distinctive metabolism of cancer, preclinically developed tumor probes may modulate cancer metabolism to reveal novel targetable vulnerabilities.

## 6 Conclusion

Hyperpolarized MRI theranostics may serve as an effective approach for cancer therapy as well as metabolic diagnosis. By providing real-time, non-invasive insights into tumor metabolism, this approach facilitates early detection, personalized treatment planning, and monitoring of therapeutic efficacy. By effectively integrating treatment strategies, this approach can be used both for diagnosis and also to intelligently enhance cancer treatment efficacy. Continued research and development are crucial to fully realize the potential of hyperpolarized MRI in oncology for future clinical applications.

## Data Availability

The original contributions presented in the study are included in the article/supplementary material. Further inquiries can be directed to the corresponding author.

## Glossary

2-HG: 2-hydroxyglutarate
2OGDD: 2-oxoglutarate dependent dioxygenase
ADP: adenosine diphosphate
α-KG: alpha-ketoglutaric acid
αKGDD: alpha-ketoglutarate dependent dioxygenase
ATP: adenosine triphosphate
CIC: mitochondrial citrate carrier
CSI: chemical shift imaging
CT: computed tomography
DNA: deoxyribonucleic acid
DNP: dynamic nuclear polarization
EMT: epithelial-mesenchymal transition
EPR effect: enhanced permeability and retention effect
FDA: Food and Drug Administration
GDH: glutamate dehydrogenase
GLS: glutaminase
HAP: hypoxia-activated prodrug
IDH: isocitrate dehydrogenase
IGRT: image-guided radiotherapy
IMRT: intensity modulated radiation therapy
k_PL_: pharmacokinetic conversion rate for pyruvate-to-lactate flux
Lac/Pyr: lactate to pyruvate ratio
MRI: magnetic resonance imaging
MR-LINAC: magnetic resonance linear accelerator
NAD^+^: oxidized form of nicotinamide adenine dinucleotide
NADH: reduced form of nicotinamide adenine dinucleotide
NADP^+^: oxidized form of nicotinamide adenine dinucleotide phosphate
NADPH: reduced form of nicotinamide adenine dinucleotide phosphate
MRSI: magnetic resonance spectroscopic imaging
NMR: nuclear magnetic resonance
OCR: oxygen consumption ratio
PET: positron emission tomography
PHIP: parahydrogen-induced polarization
pO_2_: partial pressure of oxygen
PPAT: phosphoribosyl pyrophosphate amidotransferase
ROS: reactive oxygen species
SABRE: signal amplification by reversible exchange
SASP: senescence-associated secretory phenotype
SCC: squamous cell carcinoma
SLC: solute carrier
TCA cycle: tricarboxylic acid cycle
TME: tumor microenvironment
VMAT: volumetric modulated arc therapy.
